# Transcriptome Analysis Reveals Distinct Patterns of Long Noncoding RNAs in Heart and Plasma of Mice with Heart Failure

**DOI:** 10.1371/journal.pone.0077938

**Published:** 2013-10-29

**Authors:** Danhua Li, Geng Chen, Jichun Yang, Xiaofang Fan, Yongsheng Gong, Guoheng Xu, Qinghua Cui, Bin Geng

**Affiliations:** 1 Department of Physiology and Pathophysiology, Peking University School of Basic Medical Sciences, Beijing, China; 2 Department of Biomedical Informatics, Peking University School of Basic Medical Sciences, Beijing, China; 3 Institute of Systems Biomedicine, Peking University, Beijing, China; 4 MOE Key Lab of Molecular Cardiovascular Science, Peking University, Beijing, China; 5 Institute of Hypoxia Medicine, Wenzhou Medical University, Wenzhou, Zhejiang Province, China; Maastricht University Faculty of Health, Medicine, and Life Sciences, The Netherlands

## Abstract

**Objective:**

To assess the global changes in and characteristics of the transcriptome of long noncoding RNAs (LncRNAs) in heart tissue, whole blood and plasma during heart failure (HF) and association with expression of paired coding genes.

**Methods:**

Here we used microarray assay to examine the transcriptome of LncRNAs deregulated in the heart, whole blood, and plasma during HF in mice. We confirmed the changes in LncRNAs by quantitative PCR.

**Results:**

We revealed and confirmed a number of LncRNAs that were deregulated during HF, which suggests a potential role of LncRNAs in HF. Strikingly, the patterns of expression of LncRNA differed between plasma and other tissue during HF. LncRNA expression was associated with LncRNA length in all samples but not in plasma during HF, which suggests that the global association of LncRNA expression and LncRNA length in plasma could be biomarkers for HF. In total, 32 LncRNAs all expressed in the heart, whole blood and plasma showed changed expression with HF, so they may be biomarkers in HF. In addition, sense-overlapped LncRNAs tended to show consistent expression with their paired coding genes, whereas antisense-overlapped LncRNAs tended to show the opposite expression in plasma; so different types of LncRNAs may have different characteristics in HF. Interestingly, we revealed an inverse correlation between changes in expression of LncRNAs in plasma and in heart, so circulating levels of LncRNAs may not represent just passive leakage from the HF heart but also active regulation or release of circulatory cells or other cells during HF.

**Conclusions:**

We reveal stable expression of LncRNAs in plasma during HF, which suggests a newly described component in plasma. The distinct expression patterns of circulatory LncRNAs during HF indicate that LncRNAs may actively respond to stress and thus serve as biomarkers of HF diagnosis and treatment.

## Introduction

In analyses of the human transcriptome, most transcripts have little or no protein-coding capacity but rather are noncoding RNAs [1], which adds novel content to traditional protein-centric molecular biology [2]. MicroRNAs (miRNAs), a class of small noncoding RNAs, are critical in biology and medicine [3]. However, long noncoding RNAs (LncRNAs), defined as noncoding RNA molecules greater than 200 nt, represent most of the noncoding RNAs but remain among the least well understood [4],[5].

When LncRNAs were discovered, they were considered not to have important function [5] because of their low conservation, low expression level and high tissue specificity [5]–[7]. In recent years, a number of LncRNAs have been found to have important and diverse functions [8], [9]. LncRNA-related dysfunction has been found to play critical roles in various diseases [10], [11], including cancers [12], cardiovascular diseases [2], and neurodegeneration diseases [13]. Depending on the LncRNA and disease database (LncRNA Disease [14]), LncRNAs are associated with more than 150 diseases. Therefore, LncRNAs are becoming important biological molecules for understanding the mechanisms of disease and for exploring biomarkers for disease diagnosis and treatment.

Heart failure (HF) is a complex clinical syndrome that causes high morbidity and mortality [15], [16]. HF has a complex genetic basis [17], [18], and a number of biological molecules are potential biomarkers [19] or therapeutic targets [20]. However, these molecules are protein-centric, and although they have been associated with benefit for patients with HF, the main problems of HF are still far from being solved.

Recent studies have reported that miRNAs are involved in the pathophysiologic aspects of HF and could be novel biomarkers of HF [21], so revealing a role for noncoding RNAs may help in HF diagnosis and treatment. However, the role of LncRNAs in HF is still unknown. In the present study, we used microarray assay and identified a number of LncRNAs that are deregulated in heart tissue, whole blood, or plasma during HF in a mouse model. Moreover, we reveal a stable global expression of LncRNAs in plasma. Strikingly, the expression patterns of LncRNA in HF plasma differed from and even contrasted with those in heart tissue, whole blood, and normal plasma. LncRNAs play roles in HF and, as molecules in HF plasma, may represent a novel approach for HF diagnosis and treatment.

## Materials and Methods

### Materials

Male C57BL/6J mice (18–20 g) were provided by the Animal Department, Health Science Center, Peking University. All animal care and experimental protocols complied with the Animal Management Rules of the Ministry of Health of the People’s Republic of China and the guide for the Care and Use of the Laboratory Animals of Peking University. The protocol was approved by the Laboratory Animal Management Committee of Peking University (Permit no.: 2012-0013). All animals were housed in SPF class condition and were killed under sodium pentobarbital anesthesia. TRIZOL reagent was from Invitrogen (NY, USA). RNeasy minicolumn was from Qiagen (Valencia, CA). The GoScript Reverse Transcription System (cDNA synthesis kit) and Go Taq qPCR Master Mix (SYBR green assay) were from Promega (Madison, WI). Other chemicals and reagents were of analytical grade.

### HF Model

The acute HF mouse model was induced by subcutaneous injection of isoproterenol (40 mg/kg/day) for 1 week [22] in 50 mice. Another 50 mice were injected with physiological levels of saline (0.2 mL/day) as a control. At the end of the experiment, 10 mice in each group were anesthetized with pentobarbital sodium (30 mg/kg, intraperitoneal administration). Hemodynamic parameters including heart rate, left ventricular systolic pressure (LVSP), left ventricular end diastolic pressure (LVEDP), maximal left-ventricular pressure development (LV+/−dp/dtmax) were measured by use of an intraventricular catheter from the right carotid artery linked to a Powerlab (4S, Australia). At the end of the experiment, the mice were killed, hearts were quickly removed and paraffin slices were prepared. Hematoxylin and eosin (H&E) staining was used to assess myocardial injury.

### RNA Extraction

Another 10 treated mice were killed and hearts were quickly removed and rinsed with cold sterile phosphate buffered saline (PBS; prepared with DEPC water), about 20 mg heart tissues was collected and stored in liquid nitrogen. Five samples of about 100 mg heart tissues were collected, and total RNA was extracted by use of TRIZOL reagent (Invitrogen, NY, USA). Other heart tissues were collected, and total RNA was extracted, then reverse-transcribed to cDNA for quantitative PCR analysis.

Whole blood was collected by enucleation of eyeballs. A total of 100 µL whole blood from each mouse was transferred to a tube and 1 mL whole blood was collected. Total RNA of the whole blood sample was isolated by standard one-step phenol extraction methods by use of a whole-blood RNA extraction kit (Applygen, Beijing).

Plasma was obtained by centrifugation of EDTA-blood at 1600 g for 10 min, and supernatant was centrifuged at 12000 g for 5 min at 4°C. In total, 1.6 mL clean plasma (from 10 mice) was filtered through a 0.22-µm filter (MILLEXGV; Millipore), then mixed with 2 mL TRIZOL reagent and 0.4 mL chloroform. The mixture was centrifuged at 12000 g for 5 min at 4°C, then the aqueous layer was transferred to a new tube, 1.5 mL 70% ethanol was added, then applied to an RNeasy minicolumn (Qiagen) according to the manufacturer’s recommendations. After DNase digestion, total RNA was eluted with 20 µL RNase-free water and stored in liquid nitrogen [23].

### Microarray Analysis of LncRNA and mRNA Expression

Total RNA from heart, whole blood, and plasma from five pairs of mice including five mice with HF and five mice of normal control was mixed as biological replicates, respectively. Total RNA from each sample was then quantified by use of NanoDrop ND-1000 (Thermo Fisher Scientific Inc.). The expression profiles of mouse genome-wide LncRNAs (File S1) were detected using Arraystar Mouse LncRNA Microarray v2.0, which also detected the expression profiles of mouse genome-wide protein-coding transcripts (File S2) at the same time. For microarray analysis, Agilent Feature Extraction software (version 11.0.1.1) was used to analyze the acquired array images. Quantile normalization and subsequent data processing were performed using the GeneSpring GX v11.5.1 software package (Agilent Technologies). After quantile normalization of the raw data, LncRNAs that at least 2 out of 2 samples have flags in Present or Marginal (“All Targets Value”) were chosen for differentially expressed LncRNAs screening. To identify differentially expressed LncRNAs, we performed a Fold Change filtering between the paired samples (HF vs. normal control) (Fold Change > = 2.0). The threshold for upregulation was fold change ≥2.0 and downregulation ≤0.5. We have submitted the microarray data to Gene Expression Omnibus (accession number: GSE48885).

### Quantitative PCR Analysis

cDNA samples were prepared from total RNA of heart tissues, whole blood and plasma by reverse transcription. In total, 28 LncRNAs were analyzed by SYBR green I dye-based detection with specific primer sequences (Table S1). The relative expression of LncRNAs was determined by the 2^−ΔΔCt^ method with housekeeping gene-GAPDH expression to normalize the data.

### Statistical Analysis

For microarray data, to identify differentially expressed LncRNAs, we performed a Fold Change filtering between the paired samples (HF vs. normal control) (Fold Change > = 2.0). We estimated the relationship between expression profiles of the six samples using Spearman’s correlation. We used multiple dimensional scaling (MDS) to map the six samples on a 2-D Euclidian space based on the similarity of their expression profiles. In addition, Fisher’s exact test was used to compare the relationship between differential expression of LncRNAs and their genomic neighbor mRNAs. For quantitative PCR data, we used unpaired *t* test to compare the expression of LncRNAs between HF samples and control samples. P<0.05 was considered statistically significant. All the above analysis was performed using R, a free statistical software. The clustering analysis of LncRNA length-expression relationships between samples and LncRNAs was performed by the software Cluster 3.0 and the heatmap of clustered result was generated by the software TreeView.

## Results

### Local Changes of LncRNAs in Acute HF

**Figure 1:**
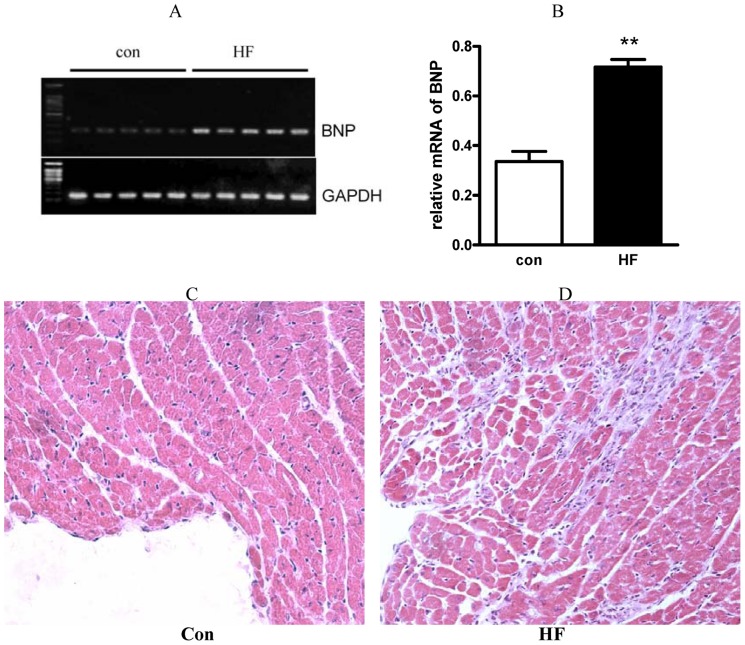
Detection of biomarkers of heart failure by BNP mRNA and necrosis of myocardial tissues. (A): BNP and housekeeping gene GAPDH gene transcription were amplified by RT-PCR assay, then the PCR productions were separated in a 1.5% agarose gel, and stained with ethidium bromide. (B): Relative BNP mRNA expression (relative to that of GAPDH) were quantified by analysis of bands density. The heart of control mice (C) and mice with heart failure (D) were performed hematoxylin and eosin staining, and local necrosis in endocardium, hyperplasia of fibroblasts were showed in heart failure heart. **, p<0.01 compared with control.

Isoproterenol induces continuous activation of β-adrenergic receptor, then increases energy consumption in cardiomyocytes, thus causing insufficient relative local energy and myocardial cell injury as with ischemic alterations and HF. Here, we confirmed the isoproterenol-induced HF in mice as elevated mRNA level of brain natriuretic protein (a molecular marker of HF; Fig. 1A and B), local necrosis in endocardium, hyperplasia of fibroblasts (Fig. 1C and D), and impaired cardiac pump function evidenced by lowered LV±dp/dt_max_ and increased LVEDP (Table 1, P<0.01).

**Table 1:** The changes of hemodynamics.

	HR(beats)	LVSP(mmHg)	LVEDP(mmHg)	LV+dp/dt_max_(mmHg/s)	LV−dp/dt_max_(mmHg/s)
Con	532±32	112±12	4.67±2.2	5563±321	5322±339
HF	546±47	108±21	16.8±5.4**	3214±485**	2647±517**

Data are mean±SD. Con: control, HF: heart failure, HR: heart rate, LVSP: left ventricular systolic pressure, LVEDP: left ventricular end diastolic pressure, LV+dp/dt_max_: the maximal left-ventricular systolic pressure development, LV**−**dp/dt_max_: the maximal left-ventricular diastolic pressure development.

** *P*<0.01 versus control.

**Figure 2:**
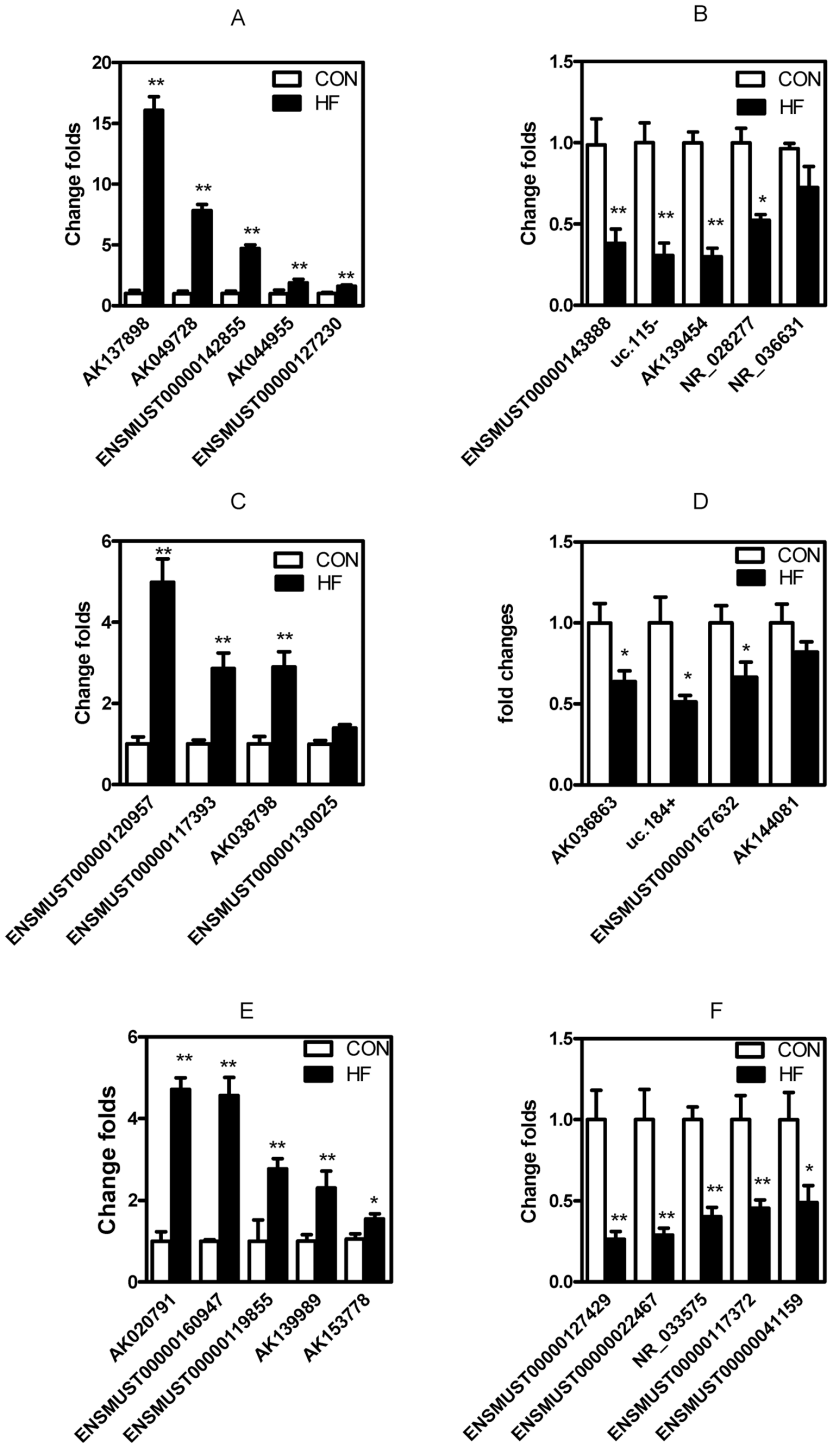
Screen for expression of deregulated long noncoding RNAs (LncRNAs) by quantitative RT-PCR assay. Five upregulated (A) and downregulated (B) LncRNAs in the heart, four upregulated (C) and downregulated (D) LncRNAs in the whole blood, five upregulated (E) and downregulated (F) LncRNAs in the plasma (E and F) were assayed by quantitative RT-PCR assay and using GAPDH housekeeping gene to normalize these data. *, **, p<0.05, p<0.01 compared with control.

In this acute HF model, we found 518 LncRNAs upregulated and 908 downregulated (Table S2) by high-output microarray analysis. Quantitative-PCR identified the relative expression of 5 significantly upregulated and 5 downregulated LncRNAs from 10 HF mice. Five LncRNAs were upregulated and 4 downregulated in HF hearts as compared with control hearts (all P<0.05; Fig. 2A and B).

### Changes in Expression of Circulatory LncRNAs in Acute HF

Plasma is an essential part of extracellular fluid contributing to balance of homeostasis. Disordered homeostasis plays a vital role in the pathogenesis of HF. HF-induced circulatory dysfunction accelerates homeostasis disorder. Thus, we hypothesized that LncRNAs might act as biomarkers of HF. We analyzed changes in expression of LncRNAs in whole blood and clean plasma. Microarray analysis revealed 1619 upregulated and 1582 downregulated LncRNAs in plasma during HF. Whole blood showed 1139 upregulated and 1506 downregulated LncRNAs (Table S3). We also identified the relative expression of some LncRNAs in whole blood (Fig. 2C and D) and plasma (Fig. 2E and F). This is the first report of LncRNAs globally existing in plasma. More intriguingly, AK143260 (Bvht), an LncRNA well known to be required for cardiovascular lineage commitment in embryogenesis [24], was significantly downregulated in plasma of HF mice (Table S3). This finding further indicates that LncRNAs represent a novel component of plasma and could be biomarkers for diseases.

### Characteristics of LncRNAs with Changed Expression in HF

**Figure 3:**
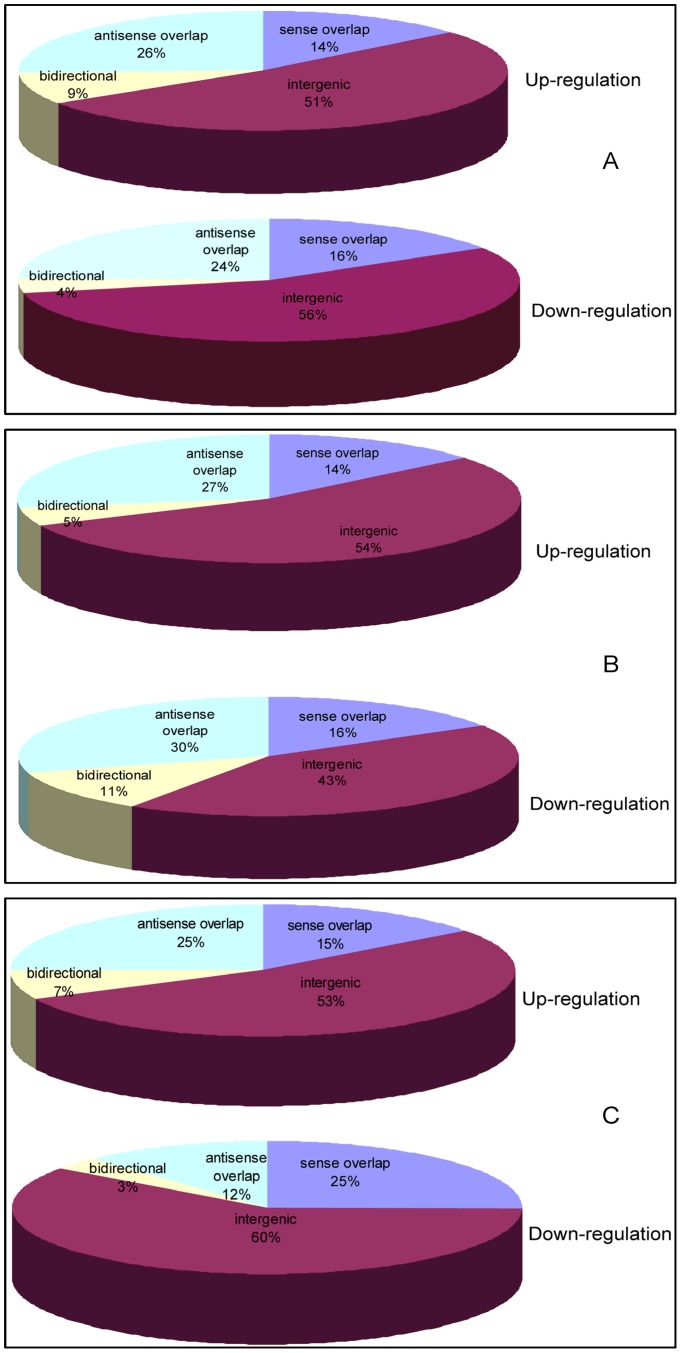
Distribution of various classes of LncRNAs in changed LncRNAs. We analyzed the ratio of four classes (sense overlap LncRNAs, antisense overlap LncRNAs, bidirectional LncRNAs, and intergenic LncRNAs) in total changed LncRNAs in the heart (A), the whole blood (B) and the plasma (C) during heart failure.

Structurally different RNAs engage in diverse mechanisms that lead to different regulatory outcomes. The primary sequences of most LncRNAs are not conserved and their function is still unclear. Some of the LncRNAs converge on chromatin to silence multiple genes located on the overlapping and non-overlapping sides. Thus, we analyzed the association of these LncRNAs with coding genes and classified them as sense-overlapping (LncRNA exon overlaps with a coding transcript exon on the same genomic strand), intronic (LncRNA intron overlaps the coding transcript intron on the same genomic strand), antisense-overlapping (LncRNA exon is transcribed from the antisense strand and overlaps with a coding transcript exon), non–antisense-overlapping (LncRNA is transcribed from the antisense strand without sharing overlapping exons), bidirectional (LncRNA is oriented head to head with a coding transcript within 1000 bp), intergenic (no overlapping or bidirectional coding transcripts). We found evidence of intronic and non-antisense overlapping for LncRNAs with changed expression in the heart (Fig. 3A), whole blood (Fig. 3B), and plasma (Fig. 3C) with HF. About 50% of LncRNAs were intergenic, about 40% were sense- and antisense-overlapping and fewer than 10% were bidirectional.

**Figure 4:**
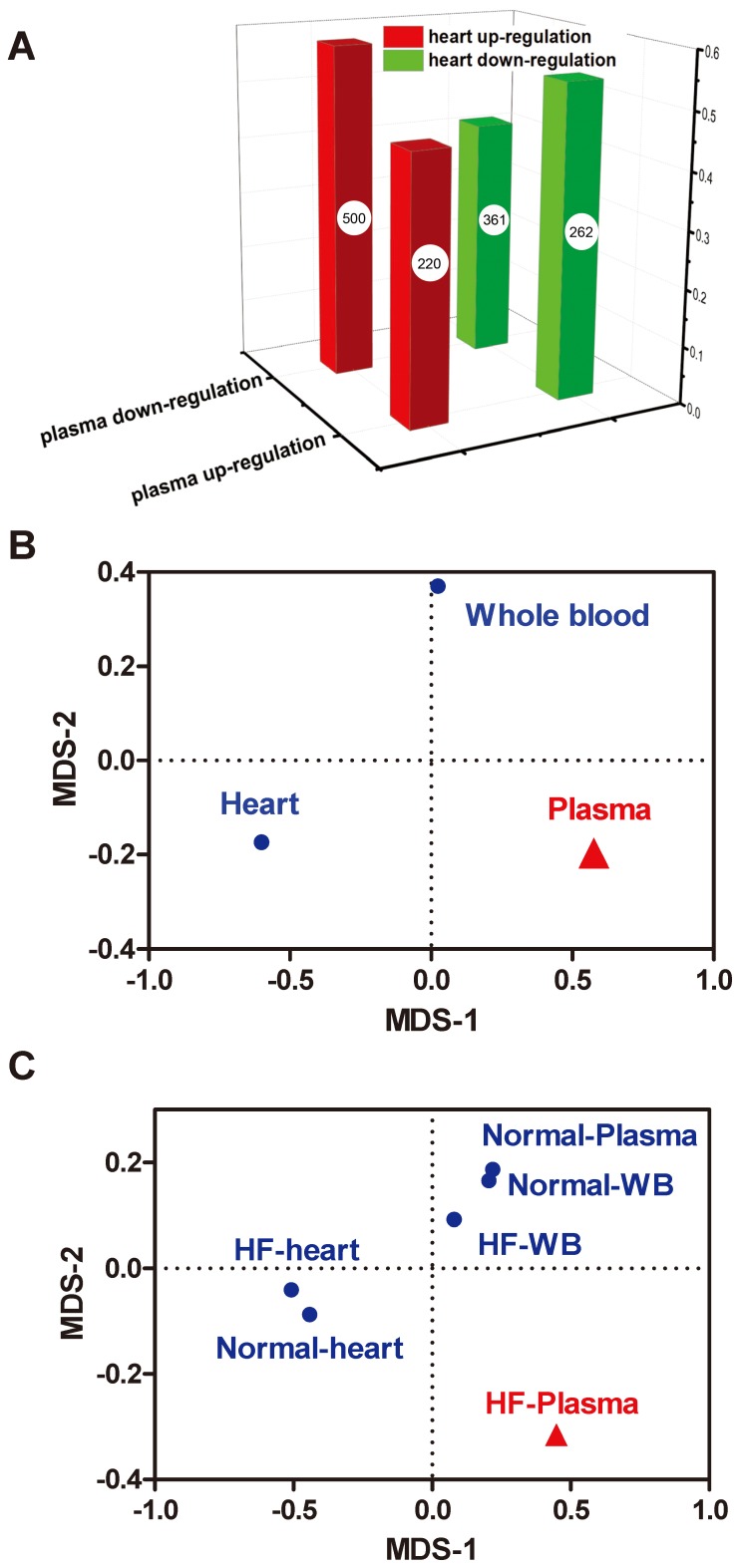
Relationships of deregulated LncRNAs among heart, whole blood, and plasma. The plasma tends to show inverse tendency with the heart (A). That is, LncRNAs upregulated (downregulated) in heart tend to be downregulated (upregulated) in plasma. The relationship among heart, whole blood, and plasma based on multiple dimensional scaling (MDS) coordinates of their correlation distance of deregulated fold was shown in (B). The relationship among heart, whole blood, and plasma based on multiple dimensional scaling (MDS) coordinates of their correlation distance of expression profile was shown in (C).

We next evaluated the co-changing of the expression difference of LncRNAs between any two samples for the six samples using Spearman’s correlation analysis. More strikingly, we found an inverse correlation between the fold change of LncRNAs in plasma and heart (R = −0.24, P = 2.2e-16, Spearman’s correlation), in that LncRNAs upregulated in the heart during HF tend to be downregulated in plasma, and vice versa. In contrast, LncRNAs upregulated in plasma tend to be downregulated in heart, and LncRNAs downregulated in plasma tend to be upregulated in heart (P = 7.7e-6, Fisher’s exact test, Fig. 4A). To visualize these relationships, we used these data to further map the findings for heart, whole blood, and plasma into a 2-D Euclidian space by multiple dimensional scaling (MDS) analysis. The pattern in plasma clearly differed from that in heart (Fig. 4B). The negative correlation between the fold change in expression of LncRNAs in plasma and heart revealed that plasma LncRNAs might be not the result of simple leakage or passive results of HF but could be actively secreted from heart and other tissues.

We wondered if it was the normal plasma or the HF plasma that contributed to the contrasting pattern of LncRNA deregulation in plasma and heart during HF. For this purpose, we investigated relationships between the LncRNA expression profiles of normal heart, HF heart, normal whole blood, HF whole blood, normal plasma, and HF plasma with MDS mapping of correlation data. The result showed that correlations for HF plasma with other samples were lower than for other comparisons. As well, MDS analysis revealed that the expression of LncRNAs in HF plasma was distinct from that in other samples (Fig. 4C).

**Figure 5:**
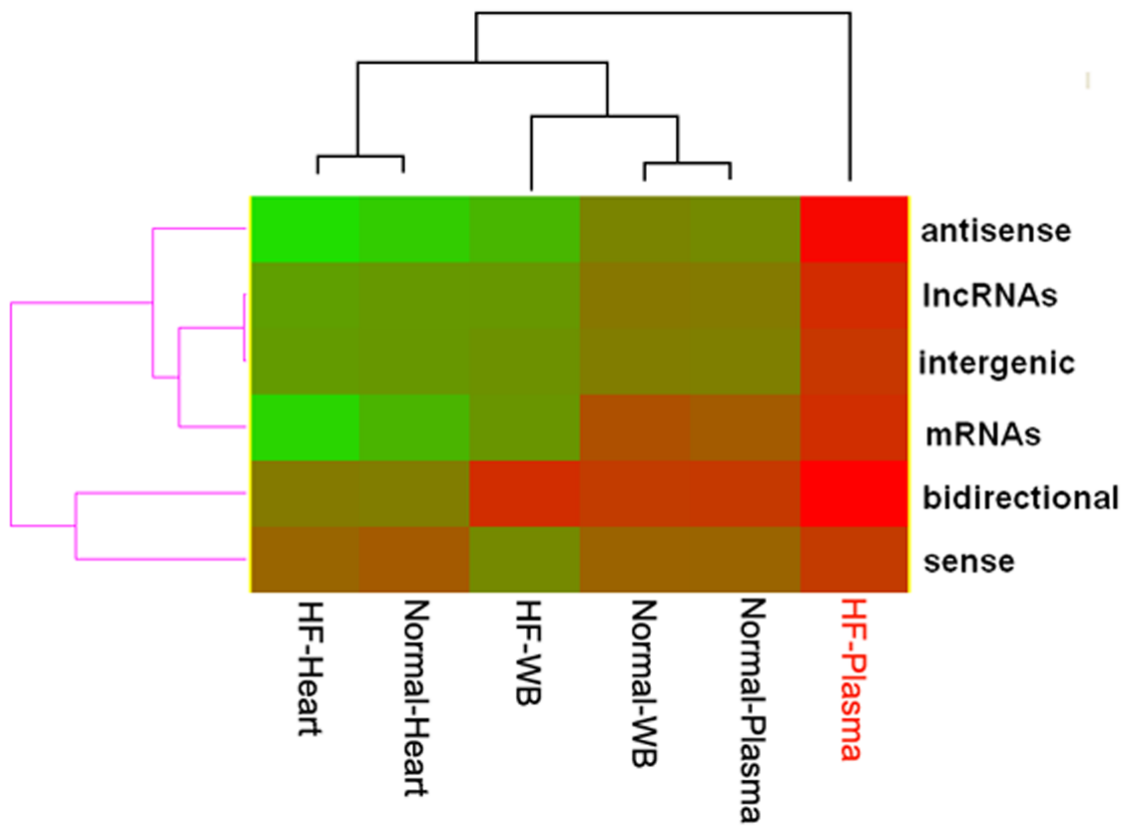
Bi-clustering heatmap of the relationship between LncRNA length and expression level across LncRNAs and samples. There are significant negative correlations between LncRNA length and expression level for all classes of LncRNAs in HF heart, normal heart, HF whole blood (WB), normal WB, and normal plasma. However, the correlation was significantly decreased and even disappeared in HF plasma.

It is known that gene length is negatively correlated with gene expression [25]. For LncRNAs, here we also found negative correlations between LncRNA length and expression (Table 2). The other five samples but the HF plasma all showed significantly negative correlation between LncRNA length and LncRNA expression level. However, the correlation between LncRNA length and expression level in HF plasma decreased greatly and even disappeared, especially for LncRNAs that are antisense or bidirectional molecules of protein-coding genes (Table 2, Fig. 5). This is the first report of a link between greatly decreased or disappeared correlation between molecule length and expression and pathogenesis. Our findings may have clinical significance because of the discrimination of physiologic and pathologic features by evaluating the global correlation between LncRNA length and expression in plasma.

**Table 2:** Correlation between LncRNA length and expression in 6 normal and heart failure (HF) samples from mice.

*Length*	*HF Heart*	*Normal Heart*	*HF WB*	*Normal WB*	*HF Plasma*	*Normal Plasma*
Total	–0.17	–0.17	–0.17	–0.13	**–0.05**	–0.17
P value	<2.2e-16	<2.2e-16	<2.2e-16	<2.2e-16	**1.6e-8**	<2.2e-16
Antisense	–0.24	–0.22	–0.207	–0.15	**–0.01**	–0.24
P value	<2.2e-16	<2.2e-16	<2.2e-16	<2.2e-16	**0.66**	<2.2e-16
Bidirectional	–0.13	–0.14	–0.05	–0.07	**0.01**	–0.06
P value	3.8e-5	2.5e-5	0.13	0.04	**0.84**	0.05
Sense	–0.11	–0.10	–0.15	–0.11	**–0.07**	–0.11
P value	6.1e-9	2.3e-7	3.1e-15	1.2e-8	**6.5e-4**	8.2e-9
Intergenic	–0.17	–0.17	–0.16	–0.14	**–0.06**	–0.14
P value	<2.2e-16	<2.2e-16	<2.2e-16	<2.2e-16	**1.1e-6**	<2.2e-16

Data are correlation coefficients (Spearman correlation) and p levels.

LncRNAs, long non-coding RNAs; WB, whole blood.

### Possibility of LncRNA as a Biomarker in HF

To screen the possibility of LncRNAs as biomarker response to HF, we analyzed the LncRNAs with changed expression among heart, plasma and whole blood during HF. As a result, we identified 32 overlapped differentially expressed LncRNAs that showed significant change in expression in all of the three samples (heart, plasma, and whole blood) during HF (Table 3). Most of the changes were consistent in plasma and whole blood, whereas about 50% of the LncRNAs showed an inverse tendency in expression between heart and plasma or whole blood. These LncRNAs might be possible biomarkers of HF.

**Table 3:** Changed LncRNAs among heart, plasma and whole blood.

*LncRNA name*	*Relationship with gene*	*Changes of LncRNA*			*Associated Gene ID*
		*Heart*	*Plasma*	*Whole blood*	
ENSMUST00000118506	intergenic	+	+	+	
ENSMUST00000121157	intergenic	+	+	+	
ENSMUST00000119799	intergenic	+	+	+	
ENSMUST00000118172	intergenic	+	+	+	
ENSMUST00000118572	intergenic	+	+	+	
uc007qxl.1	antisense overlap	+	+	−	14088
uc007qgn.1	intergenic	+	−	−	
AK082011	bidirectional	+	−	−	72401
MM9LINCRNAEXON10032−	intergenic	+	−	−	
NR_024599	intergenic	+	−	−	
ENSMUST00000120198	intergenic	+	−	−	
ENSMUST00000156391	bidirectional	−	−	+	72151
ENSMUST00000150754	intergenic	−	−	+	
ENSMUST00000152379	bidirectional	−	+	−	56397
MM9LINCRNAEXON10893+	intergenic	−	+	−	
AK155808	intergenic	−	+	−	
AK141702	intergenic	−	−	−	
AK156805	intergenic	−	−	−	
uc008uuw.1	intergenic	−	−	−	
MM9LINCRNAEXON10133−	intergenic	−	−	−	
AK015845	intergenic	−	−	−	
uc007xrw.1	intergenic	−	−	−	
NR_037610	sense overlap	−	−	−	18472
AK157007	intergenic	−	−	−	
ENSMUST00000149914	bidirectional	−	−	−	83691
AK044386	intergenic	−	−	−	
MM9LINCRNAEXON10681+	intergenic	−	−	−	
MM9LINCRNAEXON10421−	intergenic	−	−	−	
MM9LINCRNAEXON11017−	antisense	−	−	−	72543
AK156356	antisense	−	+	+	69277
uc007rjp.1	intergenic	−	+	+	
AK009126	intergenic	−	+	+	

“+” means up-regulated;

“−” means down-regulated.

### The Relationship of LncRNAs with Coding Gene Expression in HF

**Figure 6:**
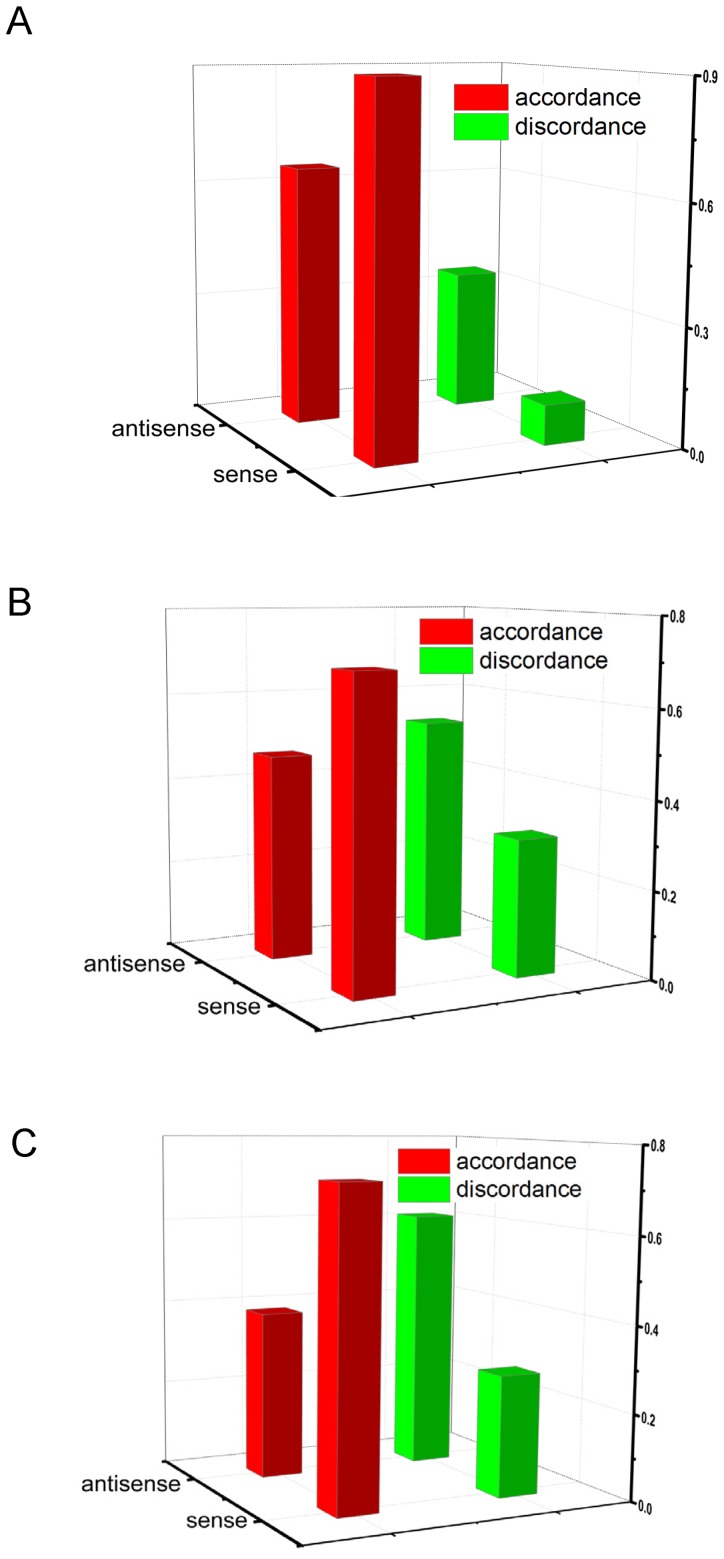
Relationship of expression-deregulation between LncRNAs and their paired protein-coding gene. We analyzed the relationship of expression of LcnRNA and their paired protein-coding gene in heart (A), plasma (B) and whole blood (C). The sense overlap LncRNAs tend to show similar tendency with their paired protein-coding genes (co-upregulation or co-downregulation). Compared with the sense overlap LncRNAs, however, the antisense overlap LncRNAs tend to show reverse tendency with their paired protein-coding genes.

Among these HF-responsive LncRNAs, 173 upregulated and 284 downregulated LncRNAs (Table S4) are overlapped with protein-coding genes (LncRNA/mRNA gene pairs). We also used mRNA microarray assay to assess changes in gene expression for the gene pairs. In mice with HF, the expression of most sense-overlapping LncRNAs agreed with that of their paired coding gene in heart tissue (OR = 4.9, P = 0.2, Fisher’s exact test) (Fig. 6A), plasma (OR = 2.5, P = 5.6e-4, Fisher’s exact test) (Fig. 6B) and whole blood (OR = 4.0, P = 2.8e-4, Fisher’s exact test) (Fig. 6C). These LncRNAs with changed expression may function in part with the expression of their paired coding gene.

## Discussion

LncRNAs are an emerging class of novel molecules defined as having RNA length >200 bases. Increasing studies have revealed that LncRNAs play an essential role in gene silencing, as trans-regulatory elements affecting multiple gene transcription, as scaffolds for histone modification, enhancers to modify gene expression, participators in genomic reprogramming involved in stem cell differentiation and nucleators to generate the dynamic assembly of the nuclear structure (reviewed in [4]). In the past decades, many works have addressed single LncRNA regulation in the pathogenesis of cancer [26], development [24], neurodegenerative disease [27], and cardiovascular diseases [2]. Recently, some works have also analyzed global changes in LncRNA expression in tissues or cells in stroke [28], adipogenesis [29], and renal clear cell carcinoma [30]. Disordered circulatory homeostasis participates in the pathogenesis, development and prognosis of disease, but the expression of LncRNAs in the circulation system and how their expression changes in disease are unclear. In this study, we globally identified the expression changes in LncRNAs in heart tissue, plasma and whole blood in mice with acute HF induced by isoproterenol, then analyzed the characteristics of LncRNAs with changed expression and possible relationship with coding genes.

In the acute HF mouse model, 518 LncRNAs were upregulated and 908 downregulated in the heart. Intriguingly, the number of LncRNAs with changed expression in plasma or whole blood was almost double that in heart tissue, which suggests that circulatory LncRNAs play an important role in pathogenesis, development and prognosis in HF. As well, large numbers of LncRNAs exist in plasma. This finding leads to the following questions: 1) Where do these LncRNAs originate? 2) Could LncRNAs secrete and how? 3) Why are these LncRNAs stable in plasma? and 4) What is their function?

Here we analyzed the characteristics of LncRNAs with changed expression during HF and found that half of them are intergenic, 40% are sense or antisense-overlapping and less than 10% are bidirectional. The number of LncRNAs expressed and with changed expression both in heart and plasma or whole blood was <10% of the total number, which suggests a different pathological response to HF in heart and blood. We analyzed the alteration in expression of LncRNAs expressed both in heart and plasma and found that the expression of LncRNAs in plasma differed from and even contrasted with that in heart or whole blood. The inverse correlation between deregulated LncRNA expression in plasma and in heart during HF is consistent with our previous observation of blood- and cancer-tissue–born miRNAs also showing an inverse relationship in expression [31]. Thus, plasma LncRNAs may not be just a leakage or passive response of the heart during HF but may be actively released from blood cell or other cells such as bone-marrow hematopoietic stem cells.

More intriguingly, the expression of LncRNAs and mRNAs in heart, whole blood even if normal plasma was negatively associated with their nucleic acid length, whereas the expression of LncRNAs in HF plasma was not correlated with length. Regulation of LncRNAs may be more complicated in HF than normal plasma and involve subtle regulatory functions to maintain the homeostasis or be a biomarker of HF. RNA expression and degradation is more quick than that of peptides or protein, and RNAs, specifically non-coding RNAs, also show multiple functions; thus, non-coding RNAs may act as a sensitive biomarker for diseases such as cancer [32], [33]. Here, we found 32 LncRNAs with expression change greater than two-fold in heart, whole blood and plasma during HF. Because most of these LncRNAs were intergenic (Table 3), their functions are little known, but they may be potential biomarkers of HF, which needs further investigation.

Here, we found that about 40% LncRNAs with changed expression during HF were sense or antisense paired coding genes in heart, whole blood and plasma, so they may regulate overlapped gene transcription. Most of the sense LncRNAs exhibited showed coordinated change in expression with that of transcripts in heart, whole blood and plasma; the expression of antisense LncRNAs agreed with changes in gene transcription in heart, with opposite changes in plasma and whole blood (Fig. 6). Our data suggest that in general, LncRNA expression agreed with that of their paired genes in HF. The results were similar with the finding of LncRNAs (>60%) localized in the promoter of their coding genes in differentiated stem cells [34]. The expression of antisense LncRNAs overlapped and was complementary to that of their paired gene; by interacting with the target transcript, LncRNAs recruit a particular RNase and promote specific cleavage and degradation of the transcript or prevent transcript recognition by RNases and protect the transcript against degradation, thereby increasing the stability of the target RNA [35]. Antisense LncRNAs also act as *cis*-acting epigenetic activators or silencers or as a *trans*-acting chromatin remodelers to regulate transcripts (reviewed in [36]). The opposite transcript regulation relationship between LncRNA and their paired gene in whole blood and plasma might explain in part the LncRNA expression discordance in plasma and whole blood with HF, with LncRNA and mRNA stability in normal plasma. These findings also imply a quick, complicated genomic regulation in heart and circulation during HF; this complex LncRNA expression and stability in plasma may suggest the possibility of LncRNAs in plasma used as biomarkers.

Given that HF is a complex disease, other biological elements at various layers of the biological network should be investigated with LncRNA functions to better understand the mechanism of HF. This study revealed that LncRNAs are an indispensable layer of molecules in HF pathogenesis. LncRNAs are potential signaling molecules with stable and distinct expression in plasma, which may have potential for HF diagnosis and treatment.

## Supporting Information

Table S1 **Primer list of LncRNAs.**
(DOC)

Table S2 **Changed LcnRNAs in heart tissues.**
(XLS)

Table S3 **Changed LcnRNAs in plasma and whole blood.**
(XLS)

Table S4 **Changed LncRNAs Homology to Protein-Coding Genes in the heart.**
(XLS)

File S1 **Profile of LncRNAs.**
(TXT)

File S2 **Profile of mRNAs.**
(TXT)
