# Supplementary material for: Transcriptome Analysis Reveals Distinct Patterns of Long Noncoding RNAs in Heart and Plasma of Mice with Heart Failure
Source: PLoS One. 2013 Oct 29;8(10):e77938. doi: 10.1371/journal.pone.0077938 (PMC3812140; doi:10.1371/journal.pone.0077938)
Supplement: Table S1 — Primer list of LncRNAs. (DOC) [file pone.0077938.s001.doc]

| **Sequence** | **Sense Primer** | **Anti-sense Primer** |
| --- | --- | --- |
| **ENSMUST00000041159** | **TTCAAGATGGAGTCCGAGTGTTC** | **TCTCACCGCAGCCACAGG** |
| **ENSMUST00000022467** | **TCACCGAGACTTCACAGAACTG** | **CCAATAGCATAGGCATTTCAGAGG** |
| **NR_033575** | **TGAGAATATGACAGGACGGCATC** | **TTCAAAGCACAGCAAACAGAGAG** |
| **ENSMUST00000117372** | **TGAATATCCCTCTGCTTGCTGAC** | **ATTGACTGTGATCTGGCGAAGG** |
| **ENSMUST00000127429** | **ACAGAGAAGAGCAGACATCAGAAG** | **GGGAAGGCACCGCATCATC** |
| **ENSMUST00000119855** | **GCGGAATAGAAGTTAGTGTGGAAC** | **TGTAGCGGAGAAGTAGCATCATC** |
| **AK020791** | **GACACACCAGAAGAGGGCATC** | **TTCCAGAGGTCCTGAGTTCAATTC** |
| **ENSMUST00000160947** | **GTCACAAGGTAGAAGAGCAAATGG** | **GGTAGTCAAGGCAGATCAGAGTC** |
| **AK139989** | **CCCTGTGAGAACCCGATAGAAAC** | **TCTGGAAGTGTATAGTGCCTGTTG** |
| **AK153778** | **GGCTGTTATGGTCTGGCTCTG** | **CCTCATTCTCCTGCCTTCATCTC** |
| **AK137898** | **CCGTGATATTCCTGTTAT** | **TTCTCTTGCCTTACTTAG** |
| **AK049728** | **GGGACTATGATGACCTGATGCTAG** | **ACTGACCTGTTGCCTAATGATCC** |
| **AK044955** | **AAGGAATCGGAGGCTTACTTCAC** | **TGGCGGCTGTTTGCTGTC** |
| **ENSMUST00000127230** | **ACTGTCAACTTGCTGGACTTAGG** | **AAGAACCCAACCCACGGAATG** |
| **ENSMUST00000142855** | **ATTGAACTTGCCTCCACCTCTG** | **GGACACTGCTCATTGCTTATCAC** |
| **ENSMUST00000143888** | **TACCGCACATTCCAGCATCTTC** | **TGGGTGGGTGGCTTTAGGG** |
| **uc.115-** | **ATTAGTTGCTGAACTCAGAGAAGG** | **TGCTTGCTAATGCTTTATTGTCTC** |
| **AK139454** | **GTCTGGGAGTTCTAAGGAGTTCTG** | **CCTGATTGACAAGTCTGTGGATTG** |
| **NR_028277** | **CACTCCCACCTCCTTCTCATTC** | **CTCAACAACGGCACCAAGATG** |
| **NR_036631** | **TTCTGGATTCTCTGGAACCTCTTG** | **CACTAGCAACTGACTCAATGATGG** |
| **ENSMUST00000120957** | **GTTAGAAAGGTGTGTGCGAATGAG** | **CCTGAAGTCTAGTTGATGGTGCTC** |
| **ENSMUST00000117393** | **CCATCTTGCTTTCATCGCTCAC** | **CCTCTGCCTTGTTTGAACCTTAG** |
| **AK038798** | **GGCTCCCTCCACTCACTTTC** | **GTGCTGAAGACCTACTGAAGAATG** |
| **ENSMUST00000130025** | **ATGGGCTGGCAGGAACAATAG** | **GAGAGGATGAGACAACGAGACAC** |
| **AK036863** | **CACCAGCAGCAGCAGAGAC** | **CTGAACACGCACGGAGAAGG** |
| **uc.184+** | **CACTGACGGGATATCTCTTTATGC** | **AAATGTATGCAGAGCACTGAAATG** |
| **ENSMUST00000167632** | **CAGGAAGAATGTCAAGAACAGGTG** | **TGTGGTTTCTGCTCTGGATCTG** |
| **AK144081** | **CTTGCCTGCCAGCGGAAG** | **TAGACTGAGGTTCGGAATCACTTG** |
